# Expression of transketolase TKTL1 predicts colon and urothelial cancer patient survival: Warburg effect reinterpreted

**DOI:** 10.1038/sj.bjc.6602962

**Published:** 2006-02-07

**Authors:** S Langbein, M Zerilli, A zur Hausen, W Staiger, K Rensch-Boschert, N Lukan, J Popa, M P Ternullo, A Steidler, C Weiss, R Grobholz, F Willeke, P Alken, G Stassi, P Schubert, J F Coy

**Affiliations:** 1 Department of Urology, University Hospital Mannheim, Theodor-Kutzer-Ufer 1-3, 68167 Mannheim, Germany; 2 Department of Surgical and Oncological Sciences, University of Palermo, Via Liborio Giuffrè, 5, 90127 Palermo, Italy; 3 Institute of Pathology, University Hospital Freiburg, Albertstr. 19, 79002 Freiburg, Germany; 4 Department of Surgery, University Hospital Mannheim, Ruprecht-Karls-University Heidelberg, Theodor-Kutzer-Ufer 1-3, 68167 Mannheim, Germany; 5 TAVARTIS GmbH, Kroetengasse 10, 64853 Otzberg, Germany; 6 Institute of Pathology, University of Palermo, Via del Vespro, 129, 90127 Palermo, Italy; 7 Department of Biostatistics, University Hospital Mannheim, Theodor-Kutzer-Ufer 1-3, 68167 Mannheim, Germany; 8 Department of Pathology, University Hospital Mannheim, Ruprecht-Karls-University Heidelberg, Theodor-Kutzer-Ufer 1-3, 68167 Mannheim, Germany; 9 R-Biopharm AG, Landwehrstrasse 54, 64293 Darmstadt, Germany

**Keywords:** pentose phosphate pathway (PPP), transketolase (TKT), transketolase-like-1 (TKTL1), aerobic glycolysis, Warburg effect, pharmacodiagnostic marker

## Abstract

Tumours ferment glucose to lactate even in the presence of oxygen (aerobic glycolysis; Warburg effect). The pentose phosphate pathway (PPP) allows glucose conversion to ribose for nucleic acid synthesis and glucose degradation to lactate. The nonoxidative part of the PPP is controlled by transketolase enzyme reactions. We have detected upregulation of a mutated transketolase transcript (TKTL1) in human malignancies, whereas transketolase (TKT) and transketolase-like-2 (TKTL2) transcripts were not upregulated. Strong TKTL1 protein expression was correlated to invasive colon and urothelial tumours and to poor patients outcome. TKTL1 encodes a transketolase with unusual enzymatic properties, which are likely to be caused by the internal deletion of conserved residues. We propose that TKTL1 upregulation in tumours leads to enhanced, oxygen-independent glucose usage and a lactate-based matrix degradation. As inhibition of transketolase enzyme reactions suppresses tumour growth and metastasis, TKTL1 could be the relevant target for novel anti-transketolase cancer therapies. We suggest an individualised cancer therapy based on the determination of metabolic changes in tumours that might enable the targeted inhibition of invasion and metastasis.

Cancer is now viewed as a disease resulting from cancer-causing genes that deregulate cellular proliferation, differentiation, and death. Genetic alterations acquired by tumours also modify their biochemical pathways, resulting in abnormal metabolism. Warburg proposed a model of tumorigenesis involving altered energy production in tumours. He identified a particular metabolic pathway in carcinomas characterised by the anaerobic degradation of glucose even in the presence of oxygen (aerobic glycolysis) that leads to the production of large amounts of lactate (known as the Warburg effect; Warburg *et al*, 1924). The relevance of aerobic glycolysis to cancer cell biology remains controversial (Garber, 2004; Gatenby and Gillies, 2004). However, the widespread clinical use of positron-emission tomography (PET) for the detection of aerobic glycolysis in tumours and recent findings have rekindled interest in Warburg's theory. Studies on the physiological changes in malignant conversion provided a metabolic signature for the different stages of tumorigenesis (Ramanathan *et al*, 2005); during tumorigenesis, an increase in glucose uptake and lactate production have been detected. The fully transformed state is most dependent on aerobic glycolysis and least dependent on the mitochondrial machinery for ATP synthesis (Ramanathan *et al*, 2005).

Other important links between cancer-causing genes and glucose metabolism have been already identified. Activation of the oncogenic kinase Akt has been shown to stimulate glucose uptake and metabolism in cancer cells and renders these cells susceptible to death in response to glucose withdrawal (Elstrom *et al*, 2004). Such tumour cells have been shown to be dependent on glucose because the ability to induce fatty acid oxidation in response to glucose deprivation is impaired by activated Akt (Buzzai *et al*, 2005). In addition, AMP-activated protein kinase (AMPK) has been identified as a link between glucose metabolism and the cell cycle, thereby implicating p53 as an essential component of metabolic cell-cycle control (Jones *et al*, 2005). These findings suggest that tumorigenesis requires derangements in known cancer-causing genes and altered energy production.

Despite this appreciation, the reason enhanced anaerobic glucose degradation occurs in tumours remains elusive. If carcinogenesis occurs by somatic evolution, then common components of the cancer phenotype result from active selection, and must, therefore, confer a significant growth advantage (Gatenby and Gillies, 2004). A prerequisite for the understanding of the altered glucose metabolism in tumours and its predicted selective growth advantage is the detailed analysis of glucose degrading pathways.

Two main pathways of glucose degradation have been identified. The observation in the 1930s that muscle extracts can catalyse the glycolysis of glucose to lactate led to the identification of the Embden-Meyerhof pathway. In this pathway, fructose-1,6-diphosphate is cleaved leading to pyruvate, which is reduced to lactate in the absence of oxygen. In addition to this pathway, glucose is also degraded by the pentose phosphate pathway (PPP).

The nonoxidative part of the PPP is controlled by thiamine- (vitamin B1) dependent transketolase enzyme reactions. Transketolase enzyme reactions of the nonoxidative part of the PPP enable oxygen-independent glucose degradation, and play a crucial role in nucleic acid ribose synthesis utilising glucose carbons in tumour cells. More than 85% of ribose recovered from nucleic acids of certain tumour cells is generated directly or indirectly from the nonoxidative pathway of the PPP (Boros *et al*, 1997). The importance of transketolases for tumour cell metabolism is underlined by the fact that the application of specific transketolase inhibitors to tumours induces a dramatic reduction in tumour cell proliferation (Rais *et al*, 1999). In addition, the activation of transketolases by application of thiamine stimulates tumour growth (Comin-Anduix *et al*, 2001). Furthermore, several natural products have been reported to inhibit transketolase enzyme activity *in vitro*, and also to inhibit cell proliferation or suppress tumour growth in mouse models or cancer patients as a result of reduction of transketolase activity (Hidvegi *et al*, 1999; Boros *et al*, 2001a, 2001b; Comin-Anduix *et al*, 2002; Jakab *et al*, 2003).

To establish a transketolase-inhibitory anticancer therapy, a high throughput screening of library compounds using recombinant human transketolase (TKT) has been performed. This approach resulted in the identification of two novel small-molecule inhibitors, which inhibit human TKT and suppress proliferation of cancer cell lines (Du *et al*, 2004).

So far, three human transketolase genes have been recognised, and the relative contributions of TKT, transketolase-like-1 (TKTL1), and transketolase-like-2 (TKTL2) to tumour-specific transketolase metabolism have not been investigated. Here, we provide evidence that TKTL1 mRNA and protein are specifically overexpressed in tumours, whereas TKT and TKTL2 expression are not upregulated. We demonstrate that TKTL1 protein is expressed in invasive tumours and predicts poor patient survival in colon and urothelial cancer. Our findings strongly indicate that overexpression of TKTL1 is responsible for the observed tumour-specific effects of transketolase enzyme reactions, and represents the basis for the observed inhibition of proliferation of cancer cells by anti-transketolase approaches.

## MATERIALS AND METHODS

### Real-time PCR and Western blot

Real-time PCR-based transcript quantification and Western blot analysis have been described previously (Coy *et al*, 2005).

### Subjects

In this study, 55 men and 15 women (median age of 60±15 years) with colon adenocarcinoma were included. All patients underwent colectomy, and 30% of colon adenocarcinomas were diagnosed as noninvasive (pTis), whereas 70% of colon adenocarcinomas were invasive at the time of diagnosis. Eleven tumours were classified as pT1, eight as pT2, 14 as pT3, and 16 as pT4, according to the UICC classification.

A total of 64 patients (median age 67.5 years) with urothelial carcinoma were enrolled in this study. Of these, 59 patients underwent treatment for urothelial carcinoma in which: 21 underwent transurethral resection for superficial bladder cancer; 22 underwent radical cystectomy; 16 underwent surgery for upper urinary tract carcinomas; and five underwent surgery for benign reasons. At the time of surgery, 14 % (8 out of 59) of the patients had lymph node metastases. Overall, 28 tumours were classified as nonmuscle-invasive (pTa, pT1 and carcinoma *in situ*) and 31 were classified as muscle-invasive (⩾pT2). Overall, 42% of the tumours showed no or only weak staining (staining score 0 or 1), 10% showed some staining (score 2), and 48% showed strong staining (score 3).

### Patient samples

Surgical resection specimens were obtained from the Department of Surgery, University Hospital Mannheim, Faculty of Clinical Medicine of Ruprecht-Karls-University Heidelberg, Germany (approval by the local Ethics Committee), and the University of Palermo, Department of Surgical and Oncological Science, Surgery Pathophysiology Section. None of the patients received neoadjuvant radiotherapy or chemotherapy. From each patient, cancerous and normal tissue was available. For RNA extraction, the specimens taken during the operation were immediately snap-frozen in liquid nitrogen and subsequently stored at −80°C until use. For immunohistochemistry (IHC), the specimens were fixed in 3.4% buffered formalin for 24 h and embedded in paraffin. Histological diagnosis was performed by three independent, experienced pathologists (A.z.H.; R.G.; G.S.).

### Immunohistochemical staining

Three to 5 *μ*m thick paraffin sections were analysed by IHC. Dewaxed sections were heated for antigen unmasking in 10 mM sodium citrate (pH 6.0) in a microwave oven for 1 min at 450 W followed by 5 min at 100 W. After rinsing in dH_2_O, inhibition of endogenous peroxidase was performed with a 5 min incubation with 3% H_2_O_2_. Endogenous avidin-biotin was blocked by the use of a commercial biotin blocking system (DAKO) for 10 min. After two washes in Tris/saline buffer (TBS), slides were incubated with 1% goat serum for 30 min to block unspecific staining. Sections were subsequently exposed to mouse anti-TKTL1 (clone JFC12T10; mouse IgG2_b_) antibody (15 *μ*g ml^−1^) or anti-Ser473 phospho-Akt (587F11; mouse IgG2_b_; Cell Signaling Technology) overnight at 4°C. The monoclonal anti-TKTL1 antibody JFC12T10 has been described previously (Coy *et al*, 2005). Slides were washed in TBS and incubated with biotinylated anti-mouse immunoglobulins for 30 min at room temperature and treated with streptavidin-peroxidase (DAKO). Staining was revealed using 3-amino-9-ethylcarbazole (AEC) substrate and counter-stained with haematoxylin (Figure 2M–T and Figure 3).

Alternatively, primary antibodies were visualised with avidin-biotinylated horseradish peroxidase complex (ABC) and diaminobenzidine tetrahydrochloride (DAB) (Elite kit; Vector Laboratories), and counter-stained with Mayer's haematoxylin (Figure 2A–L).

For scoring of TKTL1 expression, a scale from 0 to 3 was defined as: score 0 indicates 0–20%, score 1 indicates 21–50%, score 2 indicates 51–80%, and score 3 indicates >80% of the tumour cells were stained for TKTL1.

## RESULTS

### TKTL1, but not TKT or TKTL2, mRNA is overexpressed in carcinomas

Identification of genes selectively expressed or overexpressed in tumours is a crucial prerequisite for molecular diagnosis and treatment of cancer by addressing molecular targets. To identify such targets, we used the real-time PCR technique. When comparing the transcript levels from five colon cancer tissues to nontumour samples from the same patients, we initially detected a 35-fold overexpression of TKTL1 in one colon carcinoma sample. As TKTL1 is one of three highly similar transketolases encoded by three separate genes (TKT, TKTL1, TKTL2; Coy *et al*, 1996, 2005), we designed primers to specifically discriminate expression of the three transketolase genes in human carcinomas.

**Figure 1:**
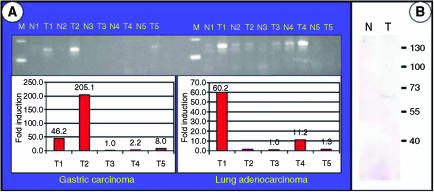
(**A**) Quantification of TKTL1 transcripts in gastric carcinoma and lung adenocarcinoma samples, and their corresponding normal tissues. In total, 15 *μ*l of the real-time PCR reaction was loaded onto a 3%-agarose gel to visualise the 150 bp TKTL1 amplification product. Expression differences between tumour and corresponding normal tissue were calculated, and are shown as fold induction in tumour sample relative to the corresponding normal sample. (**B**) TKTL1 protein expression in tumour and corresponding normal sample of a gastric carcinoma patient with a tumour-specific overexpression of TKTL1 on the transcript level was evaluated by Western blot with antibody JFC12T10. An overexpression of TKTL1 protein was observed in the tumour sample (T) compared to its corresponding normal tissue (N). Sizes of the protein marker are indicated in kDa.

Using these primers, a 79-fold overexpression of the TKTL1 gene was identified in one colon carcinoma tissue, whereas none of the tested colon carcinomas showed an overexpression of the TKT transcript. In contrast, the TKTL2 transcript was downregulated more than 10-fold in three out of five colon carcinomas (Table 1). To test whether overexpression of the TKTL1 transcript occurs in other tumour types, cDNA from five gastric and five lung adenocarcinomas and their corresponding normal tissues were analysed using the real-time PCR technique. Two of five gastric carcinomas and two of five lung adenocarcinomas had greater than 10-fold overexpression of TKTL1 (Figure 1A), whereas TKT expression was unchanged in all tested carcinoma tissues (not shown). Similar to its downregulation in colon carcinomas, TKTL2 expression was downregulated more than 10-fold in two of five lung adenocarcinomas.

In the colon carcinomas depicted in Table 1, the total amount of transketolase transcripts in four of five carcinoma tissues (Table 1; T2–T5) was lower than that in normal tissue, even in the colon carcinoma tissue with an overexpression of TKTL1 (Table 1; T5). Overexpression of TKT was not detected in any of the 54 carcinoma tissue samples tested. The only transketolase gene overexpressed in carcinoma tissue was the TKTL1 gene.

### TKTL1 protein is overexpressed in human carcinomas

**Figure 2:**
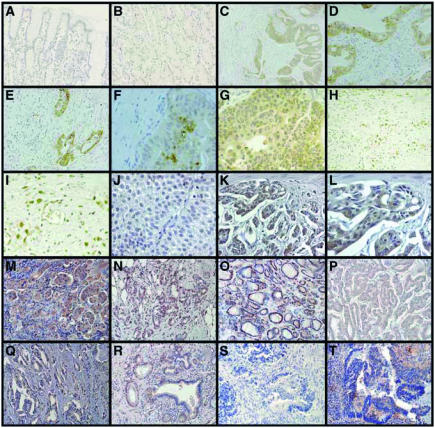
Expression of TKTL1 in normal and carcinoma tissues. Specimens of a gastric carcinoma (**C–G**) and corresponding normal tissue (**A**, **B**); (**A**, **B)** no expression of TKTL1 in normal tissue. (**C–G**) Strong cytoplasmic expression in tumour tissue, but no expression in the surrounding stroma cells. Note the elevated expression within the inner region of the tumour (**F**). (**H**, **I**) Nuclear TKTL1 expression in a poorly differentiated gastric carcinoma. (**J**) No expression of TKTL1 in a superficial, Ta bladder carcinoma. (**K**, **L**) Strong TKTL1 cytoplasmic expression in an invasive, poorly differentiated bladder carcinoma. Strong TKTL1 upregulation in carcinomas of the lung (non-small-cell lung carcinomas; **M**), breast (**N**), thyroid (follicular thyroid carcinoma (**O**), papillary thyroid carcinoma (**P**)), prostate (**Q**), and pancreas (**R**). No expression of TKTL1 in a noninvasive colon carcinoma (**S**), and strong expression in an invasive colon carcinoma (**T**). Anti-TKTL1 was revealed by diaminobenzidine tetrahydrochloride (DAB; brown staining) (**A–L**) and 3-amino-9-ethylcarbazole (AEC; red staining) (**M–T**).

In order to determine TKTL1 protein expression levels in human carcinomas, we performed IHC on 1030 human carcinomas derived from 16 different epithelial tumour entities using a monoclonal antibody (JFC12T10) that specifically detects TKTL1 (Coy *et al*, 2005). A gastric carcinoma specimen that we found to have 1000-fold overexpression of TKTL1 mRNA when compared to the corresponding normal tissue showed a strong overexpression of the TKTL1 protein on Western blot level (Figure 1B) as well as a strong TKTL1 immunoreactivity on paraffin sections (Figure 2C–G). Mainly cytoplasmic expression was detected. TKTL1 expression was restricted to tumour cells, and the surrounding stromal tissue showed no staining. In the corresponding normal tissue, no staining was detected (Figure 2A and B). Immunohistochemical analysis of two gastric carcinoma samples without overexpression of TKTL1 transcript did not reveal TKTL1 protein expression. These results demonstrate a strong correlation of TKTL1 mRNA and protein expression.

In some cases of undifferentiated gastric carcinoma, strong nuclear expression was observed (Figure 2H and I). Analysis of bladder carcinomas showed absence of TKTL1 reactivity in superficial, nonmuscle-invading tumours (Figure 2J), whereas invasive tumours showed immunoreactivity (Figure 2K and L). Non-small-cell lung carcinomas (NSCLC) (Figure 2M), breast carcinomas (Figure 2N), follicular thyroid carcinomas (FTC) (Figure 2O), papillary thyroid carcinomas (PTC) (Figure 2P), prostate carcinomas (Figure 2Q), pancreas carcinomas (Figure 2R; and undifferentiated thyroid (UTC), ovarian, cervix, rectal, and kidney carcinomas (not shown) also showed strong upregulation of TKTL1. Similar to bladder carcinomas, no or weak reactivity for TKTL1 was observed in noninvasive colon carcinomas (Figure 2S), whereas in invasive tumours, strong TKTL1 staining was detected (Figure 2T). All histological variants of thyroid cancer (FTC, Figure 2O; PTC, Figure 2P; UTC, not shown) revealed abundant TKTL1 expression within the cytoplasm. Interestingly, the majority of nuclei were stained in FTC (Figure 2O), whereas in PTC (Figure 2P), and UTC, (not shown) only few nuclei were TKTL1-positive. Non-small-cell lung cancer cells were strongly positive for TKTL1 within the cytoplasm, whereas nuclear staining was absent (Figure 2M).

### Immunohistochemical localisation of TKTL1 protein and activated Akt

**Figure 3:**
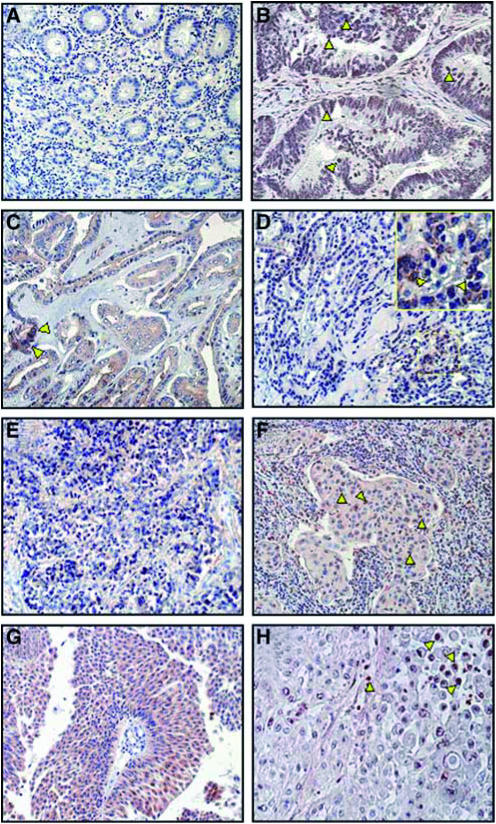
Upregulation of phosphorylated Akt (p-AKT) in epithelial tumours. Immunohistochemical analysis of p-AKT on paraffin-embedded sections from normal colon as negative control (**A**), colon cancer (**B**), papillary (PTC) (**C**), follicular (FTC) (**D**), undifferentiated thyroid carcinoma (UTC) (**E**), non-small-cell lung cancer (NSCLC) (**F**), bladder cancer (**G**), and prostate cancer (**H**) (red staining). All the different types of cancer examined showed strong staining for p-Akt (cytoplasmic, nuclear, or both cytoplasmic and nuclear) while normal tissues showed no or very weak staining (**A**). A mainly nuclear localisation of p-Akt was been detected in colon, lung, and prostate carcinomas (**B**, **F**, **H**; yellow arrowheads). In all the histological variants of thyroid and bladder cancers, strong cytoplasmic staining was detectable (**C–E**, **G**) and only few nuclei in PTC and FTC samples were positive for p-Akt (**C**, **D**; yellow arrowheads).

Recent studies have demonstrated that activated Akt exerts a direct influence on glucose metabolism leading to a dose-dependent stimulation of aerobic glycolysis (Elstrom *et al*, 2004), and to an inhibition of *β*-oxidation of fatty acids (Buzzai *et al*, 2005). Therefore, we investigated whether tumours that overexpress the TKTL1 protein also have activated Akt (phospho-Akt; p-Akt) indicative of an activated glucose metabolism and an inhibited *β*-oxidation of fatty acids. We performed IHC to detect p-Akt in thyroid, lung, colon, bladder, and prostate cancer specimens (Figure 3). All the different types of cancer examined showed strong staining for p-Akt in the cytoplasm, nucleus, and both the cytoplasm and nucleus, whereas normal tissues showed no or very weak staining (Figure 3A). In lung, colon, and prostate carcinomas, mainly nuclear localisation of p-Akt was seen (Figure 3B, F and H; yellow arrowheads), whereas in all the histological variants of thyroid cancer, and bladder cancer, strong cytoplasmic staining was seen (Figure 3C–E and G). Only few nuclei in PTC and FTC samples were positive for p-Akt (Figure 3C and D, yellow arrowheads). Activated Akt was detected in 69% of carcinomas examined, whereas 83% of tested carcinoma specimens showed overexpression of TKTL1.

### TKTL1 protein is overexpressed in invasive colon carcinomas and is associated with poor patient survival

**Figure 4:**
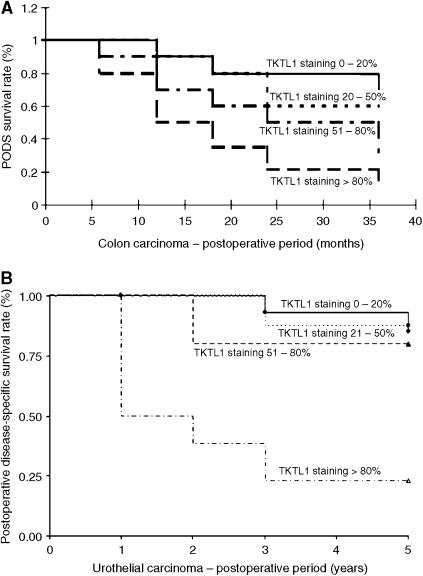
Kaplan–Meier plots demonstrating the significant correlation between TKTL1 staining intensity and survival in colon carcinoma (**A**), and in urothelial carcinoma (**B**). Scores indicate the fraction of tumour cells in each sample that stained for TKTL1 protein, as defined in the ‘Materials and methods’ section.

In order to study the correlation of TKTL1 expression on clinical–pathological parameters, a retrospective survey of surgical samples from 70 patients (55 men and 15 women, median age of 60±15 years) with colon adenocarcinoma was performed. Non-neoplastic colon tissues examined did not reveal TKTL1 expression, and noninvasive colon cancer specimens were negative or barely positive for TKTL1 staining (Figure 2S). In contrast, all invasive colon carcinomas showed TKTL1 expression (Figure 2T), in particular, 20% were classified as weakly positive for TKTL1 (score 1+), 25% as positive (score 2+), and 55% as strongly positive (score 3+). A significant correlation between TKTL1 expression and survival was found (Figure 4A).

### TKTL1 protein is overexpressed in invasive urothelial carcinomas and predicts poor patient survival

To see if the above results could be extended to other tumour types, we performed a retrospective survey of urothelial carcinomas for expression of TKTL1. Of 64 surgical cases, 59 had malignancies of the urothelial tract. The correlation between tumour stage and TKTL1 protein staining intensity was 100% score 0 in pTa superficial carcinomas, 76% score 0 or 1 and 24% score 2 or 3 in pT1 tumours. Of the muscle-invasive tumours (⩾pT2) 3% had score 0, 3% had score 1, 13% had score 2, and 81% had score 3 TKTL1 staining. There was statistically significant correlation between staging and staining intensity (Spearman correlation coefficient 0.836, confidence interval 0.76–0.92). The overall disease-specific 5-year survival rate was 47% (6 pTa, 14 pT1, 1 cis, 7 pT2). Patients dying because of tumour progression showed in 83% most intensive staining pattern of the tumours. Three patients were lost to follow-up and four patients died because of other reasons. Of 10 tumours initially found to be metastatic, nine showed strong overexpression of TKTL1 (grade 3). The correlation between staining intensity and patient survival was significant (*P*=0.001), and is shown in the Kaplan–Meier plot (Figure 4B).

## DISCUSSION

In 16 epithelial tumour entities we tested, a subgroup of tumours was found to have upregulated TKTL1 protein. This demonstrates that TKTL1 upregulation is a general phenomenon in epithelial malignancies. To determine whether TKTL1 expression correlates with clinical outcomes of colon and urothelial cancer patients, we performed a retrospective survey of surgical samples and determined the survival of patients with tumours expressing or lacking TKTL1. We found that in colon carcinomas and urothelial carcinomas, expression of TKTL1 transketolase correlated with invasiveness of tumours and poor patient survival. As it is important to have diagnostic tests to distinguish between clinically aggressive and clinically indolent forms of cancer, our findings demonstrate that the expression of TKTL1 may indicate which tumours have invasive behaviour leading to poor patient survival.

Why does TKTL1 overexpression correlate with tumour invasion? Transketolase enzyme reactions control the nonoxidative part of the PPP. Using metabolic control analysis methods and oxythiamine, Comin-Anduix *et al* (2001) demonstrated that transketolase enzyme reactions determine cell proliferation in the Ehrlich's ascites tumour model. The transketolase enzyme reactions and other reactions of the PPP allow glucose conversion to ribose for nucleic acid synthesis and generate NADPH, a reducing agent required for synthesis reactions. Both of these products of the PPP are required for growing tumour cells. In addition, the nonoxidative part of the PPP allows anaerobic glucose degradation; anaerobic conditions are often present in tumours and limit the growth of tumours. Even in premalignant lesions, which are often characterised as highly vascularised, near-zero partial pressures of oxygen are observed at distances of only 100 *μ*m from blood vessels (Gatenby and Gillies, 2004). Tumour cells that upregulate transketolase enzyme reactions can use glucose as an energy source through nonoxidative generation of ATP (Coy *et al*, 2005).

If the changes on the path toward tumorigenic conversion are seen as a Darwinian selection process conferring a selective growth advantage, activation of a nonoxidative glucose degradation pathway is likely to contribute to the individual fitness of such a tumour cell if anaerobic conditions are present. Mutations in anaerobic glucose degrading pathways allowing such a selective growth advantage would represent additional mutations leading to a fully transformed tumour phenotype. By determining physiological changes in a cell-line model of tumorigenesis, a fully transformed cell line was most dependent on aerobic glycolysis and least dependent on the mitochondrial machinery for ATP synthesis (Ramanathan *et al*, 2005). During progression to full transformation, sensitivity to oligomycin, an inhibitor of mitochondrial ATP synthase, declined progressively. No decrease in ATP levels upon treatment with oligomycin was observed in the fully transformed cell line, consistent with the predominant production of ATP by aerobic glycolysis (Ramanathan *et al*, 2005). The fully transformed cell-line was most sensitive to both 2-deoxyglucose and oxamic acid, an inhibitor of lactate dehydrogenase. The findings in this cell-line model of tumorigenesis confirm the Warburg effect and the important role of an anaerobic glucose degradation pathway for tumorigenesis.

Although the molecular and biochemical basis of this metabolic pathway has been elusive yet, both glucose usage as well as lactate production are clinically relevant. The glucose usage in tumours can be noninvasively visualised using PET with the glucose-analogue tracer, fluorodeoxyglucose (FdG). Furthermore, it has been shown that both glucose usage and lactate production represent markers indicating poor prognosis (Di Chiro *et al*, 1987; Haberkorn *et al*, 1991; Downey *et al*, 2004; Walenta *et al*, 2004). The important role of transketolase enzyme reactions for this glucose degrading and lactate producing pathway has been shown (Braun *et al*, 1997; Lee *et al*, 1998).

The PPP allows nonoxidative glucose degradation and enables a lactate-based matrix-degradation of the surrounding tissue (Stern *et al*, 2002). A lactate-based acidification of the surrounding tissue may be supplemented by the production of H_2_CO_3_ if CO_2_ is produced by the oxidative part of the PPP. Both lactate acid and H_2_CO_3_ production lead to excretion of protons and a decline in pH of the surrounding matrix. This tissue acidosis triggers a p53-mediated cell death of neighbouring healthy cells (Williams *et al*, 1999; Park *et al*, 2000). Tumour cells survive due to mutations in p53 or some other components in the apoptosis pathways. An acid-mediated tumour invasion model including promotion of angiogenesis, proteolytic cleavage of matrix proteins, and inhibition of immune response has been proposed (Gatenby and Gawlinski, 2003).

Although aerobic glycolysis leads to the above-mentioned selective growth advantages, the most important question remains unresolved. What is the energetic basis of anaerobic glucose degradation? An analysis of the contribution of different fuels and metabolic pathways in proliferating MCF7 breast cancer cells has demonstrated that 65% of the total ATP turnover is from unidentified sources (Guppy *et al*, 2002). Ramanathan *et al* (2005) have shown that the fully transformed tumour cell-line does not show a decrease in ATP levels, even if mitochondrial ATP production is inhibited. What is the source of ATP in fully transformed tumour cells when mitochondrial ATP production is not important?

While considering the above-listed advantages the nonoxidative PPP confers upon tumour cells, it is important to stress that the PPP is energetically as inefficient as the anaerobic glucose degradation via the Embden-Meyerhof pathway. When oxygen is absent, glucose degradation via both the Embden-Meyerhof pathway and the PPP leads to lactate production. Muscles perform the final mitochondrial oxidative steps of oxidative phosphorylation only if oxygen is present (Pasteur effect; Warburg *et al*, 1924). Whereas tumour tissues do not (Warburg *et al*, 1924). An unappreciated finding Warburg made was that aerobic glycolysis occurs in healthy tissues like retina and testis (Warburg *et al*, 1924). Strikingly, those healthy tissues in which Warburg detected a high aerobic glycolysis were precisely the same tissues in which a high level of TKTL1 expression has been observed (Coy *et al*, 2005). Why do tumour cells still continue to degrade glucose to lactate even in the presence of oxygen if this anaerobic pathway is energetically inefficient?

The energetic output of anaerobic glucose degradation, either based on the Embden-Meyerhof pathway or the PPP reactions as presented in text books, is worse than the energetic output of oxidative glucose degradation. Interestingly, the proposed reactions of the nonoxidative PPP are not firmly established; experimentally measured degree of ^14^C isotope labelling and its distribution in carbon atoms of fructose-6-phosphate differs from that predicted by reaction sequences (Horecker *et al*, 1954; Williams *et al*, 1987; Schenk *et al*, 1998). This discrepancy demonstrates the presence of other types of nonoxidative PPP reactions, although the molecular and biochemical basis of these reactions remain elusive.

During the evolution of higher vertebrates, mutations in the TKTL1 gene have led to an altered substrate specificity and altered enzymatic reaction kinetics. An enhanced, one-substrate reaction allowing the use of xylulose-5-phosphate as sole substrate has been detected (Coy *et al*, 2005). A TKTL1-based lactobacillae-like glucose degradation pathway has been postulated to explain the observed glucose metabolism in tumours (Coy *et al*, 2005), and such a pathway would result in enhanced glucose usage, enhanced carbonic anhydrase enzyme activity, enhanced *de novo* fatty acid synthesis, inhibition of Embden-Meyerhof glycolysis, enhanced lactate production, and mitochondria-independent ATP generation. Such postulated metabolic changes indeed have been observed in tumours. Enhanced glucose usage (Warburg *et al*, 1924; Di Chiro *et al*, 1987; Haberkorn *et al*, 1991; Downey *et al*, 2004), upregulation of carbonic anhydrase IX (Robertson *et al*, 2004), fatty acid synthase and *de novo* fatty acid synthesis upregulation (Menendez and Lupu, 2004), pyruvate kinase activity downregulation (Hardt *et al*, 2004), enhanced lactate production (Warburg *et al*, 1924; Walenta *et al*, 2004), and mitochondria-independent ATP generation (Warburg *et al*, 1924; Ramanathan *et al*, 2005; Xu *et al*, 2005) have been detected in tumours, and most of these metabolic changes are prognostic markers indicating poor patient survival.

As lactate and pyruvate regulate hypoxia-inducible gene expression independently of hypoxia by stimulating the accumulation of hypoxia-inducible Factor 1 alpha (Lu *et al*, 2002), activation of the TKTL1 pathway leading to lactate and pyruvate may also contribute to angiogenesis under normoxic conditions.

The presence of the TKTL1 pathway in tumor cells would also explain why tumor cells with an inhibited mitochondrial respiration and a concomitant enhanced aerobic glycolysis are less sensitive to induction of apoptosis by common anticancer agents, but highly sensitive to an inhibition of aerobic glycolysis (Xu *et al*, 2005). Furthermore, TKTL1-expressing tumours may derive an additional selective growth advantage if the synthesis of fatty acids is not reversed by *β*-oxidation of fatty acids. Recent studies have shown that this metabolic adaptation for an efficient growth is present in tumours. In tumours, activated Akt inhibits *β*-oxidation of fatty acids (Buzzai *et al*, 2005). If glucose is available, such a tumour cell has a selective growth advantage because produced fatty acids are not degraded; however, this metabolic change leads to absolute glucose dependence and death, in response to glucose withdrawal (Elstrom *et al*, 2004; Buzzai *et al*, 2005). The immunohistochemical analysis of expression of TKTL1 and activated Akt presented here demonstrate that the majority of TKTL1 overexpressing tumours do have activated Akt and therefore not able to perform *β*-oxidation of fatty acids.

All the observed metabolic changes in tumours are consistent with a TKTL1-based, lactobacillae-like glucose metabolism in tumours. Which of the altered enzymatic activities leading to these metabolic changes can be exploited for future cancer therapies? The most effective way to inhibit tumour proliferation should be to block the generation of energy for tumour growth. Metabolic control analysis and inhibition of transketolase enzyme reactions have already shown that tumour proliferation can be inhibited by anti-transketolase approaches (Rais *et al*, 1999; Boros *et al*, 2001a, 2001b; Comin-Anduix *et al*, 2001, 2002; Du *et al*, 2004). These successful results have been attributed to TKT transketolase. The enzymatic properties of TKTL1 (Coy *et al*, 2005), its upregulation in tumours, and the absence of upregulation of TKT and TKTL2 indicate that TKTL1 is the transketolase targeted by anti-transketolase drugs. The determination of TKTL1 expression in human malignancies may help to identify cancer patients that would benefit from an anti-transketolase cancer therapy. As a proportion of TKTL1 overexpressing tumours does not express activated Akt, cancer patients with such tumours may benefit from a concomitant inhibition of fatty acid *β*-oxidation by activation of Akt or inhibition of AMPK. Future TKTL1-based anticancer therapy may also be improved by substrate limitation through application of a ketogenic diet (Nebeling *et al*, 1995).

## Figures and Tables

**Table 1:** Fold overexpression of transketolase transcripts in colon carcinomas (T1–5) in comparison to their corresponding normal tissues

	**T1**	**T2**	**T3**	**T4**	**T5**
TKT	1	1	1	1	1
TKTL1	1	1	1	1	79
TKTL2	1	−9500	−3	−12	−375

Negative numbers indicate fold downregulation. Results are mean values of three independent experiments.
